# Case report: Suspected organizing pneumonia secondary to severe respiratory syncytial virus pneumonia in an elderly patient

**DOI:** 10.3389/fmed.2024.1394542

**Published:** 2024-07-08

**Authors:** Min Feng, Jie Zhang, Xiangrui Li, Shuai Wang, Yanxia Li, Chang Dong

**Affiliations:** ^1^Department of Respiratory and Critical Care Medicine, First Affiliated Hospital of Dalian Medical University, Dalian, China; ^2^School of Health Care Technology, Dalian Neusoft University of Information, Dalian, China

**Keywords:** respiratory syncytial virus infection, organizing pneumonia, oral ribavirin, corticosteroids, elderly patient, case report

## Abstract

Respiratory syncytial virus (RSV) usually causes acute respiratory tract infection in infants. In recent years, it has gradually become an important pathogen of lower respiratory tract infection in elderly people with an underlying disease. However, at present, the treatment of severe RSV pneumonia in adults is unclear, and organizing pneumonia (OP) after severe RSV infection has rarely been reported. We reported a 76-year-old man with multiple chronic heart and lung diseases who presented with fever, cough and progressive dyspnea. Finally, severe RSV pneumonia was diagnosed after his nasopharyngeal swabs and bronchoalveolar lavage metagenomic next-generation sequencing tests were positive for RSV. After combined treatment with oral ribavirin, intravenous immunoglobulin and corticosteroids, the patient’s condition largely resolved, and he was discharged. However, when the corticosteroids were gradually tapered, the disease relapsed twice, and the patient experienced fever and aggravated dyspnea. Despite the lack of pathological evidence, we highly suspected organizing pneumonia secondary to severe RSV pneumonia based on the typical imaging manifestations and the clinical characteristics of a good response to corticosteroids. Finally, this patient was successfully treated with a course of corticosteroids and followed up for 14 months in total.

## 1 Introduction

Respiratory syncytial virus (RSV) is one of the main pathogens causing lower respiratory tract infection which affects people of all ages, but it can be particularly severe or even fatal in infants and older adults (1). In recent years, RSV has gradually become an important respiratory infection pathogen in adults. It has been reported that 3–7% of healthy elderly people (over 65 years old) and 4–10% of high-risk adults develop RSV infection every year (2). The hospitalization mortality rate of patients with acute respiratory RSV infection is 9.1–15.9% (3–5), and it will further increase to 25 and 55% for people admitted to the intensive care unit (ICU) (6) and people with hematological malignancies (7), respectively. The risk factors for RSV infection and death in the ICU include old age, chronic heart and lung diseases, type 2 diabetes, RSV pneumonia and its complications (8).

Organizing pneumonia (OP) refers to a granuloma with loose connective tissue that occurs after a lung injury caused by incomplete absorption of cellulose in the lung. When the cause of OP is unknown, it is called cryptogenic organizing pneumonia (COP), and secondary organizing pneumonia (SOP) is usually related to known causes, including infection, drugs and radiation therapy (9). In addition, SOP after infection, including bacterial, viral, fungal and mycobacterial infections, is the most common (10–14). SOP can be related to various viral infections, including COVID-19 (15), influenza virus (16), herpes virus (17), and cytomegalovirus (18). However, SOP after severe RSV pneumonia has rarely been reported. We report a case of suspected SOP after severe RSV infection in an elderly patient and summarize the course of treatment and follow-up for 14 months.

## 2 Case presentation

**Figure 1:**
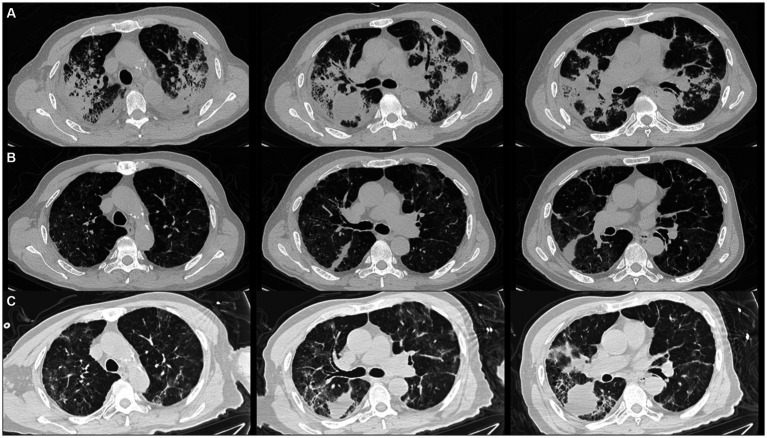
Patient’s chest computed tomography (CT) scan images at three time points. **(A)** Initial chest CT showed bilateral peribronchchial distribution of multifocal opacities and consolidations. **(B)** After taking methylprednisolone for 2 months, chest CT revealed that the bilateral opacities and consolidations were significantly absorbed. **(C)** After taking methylprednisolone for 3 months and the patient just stopped using it, chest imaging revealed that the bilateral opacities and consolidations were aggravated again.

A 76-year-old male who presented with sore throat, muscular soreness, fever, cough and dyspnea for 20 days was admitted to our respiratory ICU on February 21, 2022. He was treated with cefuroxime and levofloxacin at another hospital and then treated with meropenem combined with vancomycin for presumed bacterial infection. However, his symptoms did not improve, and he developed more severe dyspnea. His chest computed tomography (CT) demonstrated bilateral peribronchchial distribution of multifocal opacities and consolidations (Figure 1A).

The patient had smoked for more than 30 years, and his medical history included chronic obstructive pulmonary disease, diabetes, coronary heart disease and apical hypertrophic cardiomyopathy. Moreover, he had undergone radiofrequency ablation for paroxysmal atrial fibrillation 10 and 1 years ago. Upon admission, physical examination revealed that his body temperature, pulse, respiratory rate and blood pressure were 37.8°C, 102 beats/min, 30 breaths/min and 130/80 mmHg, respectively, and his oxygen saturation was as low as 79% while he was breathing ambient air. The patient had obvious cyanosis of the lips and extremities, and no dry or wet rales were heard in either lung. Arterial blood gas analysis revealed that the oxygen partial pressure was 40 mmHg under room air, indicating type I respiratory failure. C-reactive protein was 90.4 mg/L, procalcitonin and white blood cell count were normal, but the absolute lymphocyte count was as low as 0.75 × 10^9^/L. Further lymphocyte tests revealed a decreased CD3^+^ CD4^+^ T-cell count of 192 cells/μL (normal range, 368–1,632 cell/μL), indicating compromised immunity. T-cell spots indicating *Mycobacterium tuberculosis* infection were positive, the testing for *Mycobacterium tuberculosis* by polymerase chain reaction (PCR) and acid-fast staining in both sputum and bronchoalveolar lavage fluid (BALF) was negative. Extensive serum tests, including 1,3-beta-D glucan, galactomannan tests, *Aspergillus* antibody, Cryptococcus capsular polysaccharide antigen, Mycoplasma antibody, human immunodeficiency virus antibody, Epstein–Barr virus PCR, cytomegalovirus PCR, and A and B influenza virus PCR, were all negative. His brain natriuretic peptide level was 362.87 ng/L (normal range, 0–100 ng/L), and his myocardial enzymes were normal. However, the respiratory virus multiplex real-time reverse transcriptase polymerase chain reaction (PCR) of the nasopharyngeal swab specimen was positive for RSV twice but negative for A and B influenza virus, human rhinovirus, adenovirus and coronavirus. Further BALF metagenomic next-generation sequencing also revealed a positive result for RSV, with 46,671 uniquely mapped reads, which confirmed the diagnosis of RSV infection.

The patient was initially given empirical meropenem and voriconazole and intravenous injection of 80 mg/day methylprednisolone for 5 days, and his respiratory failure quickly resolved. After the diagnosis of RSV pneumonia, 0.15 g of oral ribavirin tablets three times per day and intravenous immunoglobulin were also administered in addition to 40 mg of methylprednisolone per day. The patient’s body temperature returned to normal, and his symptoms of cough and dyspnea resolved. The patient was therefore discharged and continued to receive oxygen therapy at home. The dosage of methylprednisolone was decreased to 20 mg and we decreased 4 mg of methylprednisolone every 2 weeks. At the 2-month follow-up after discharge, chest CT revealed that the bilateral opacities and consolidations were significantly reduced (Figure 1B).

**Figure 2:**
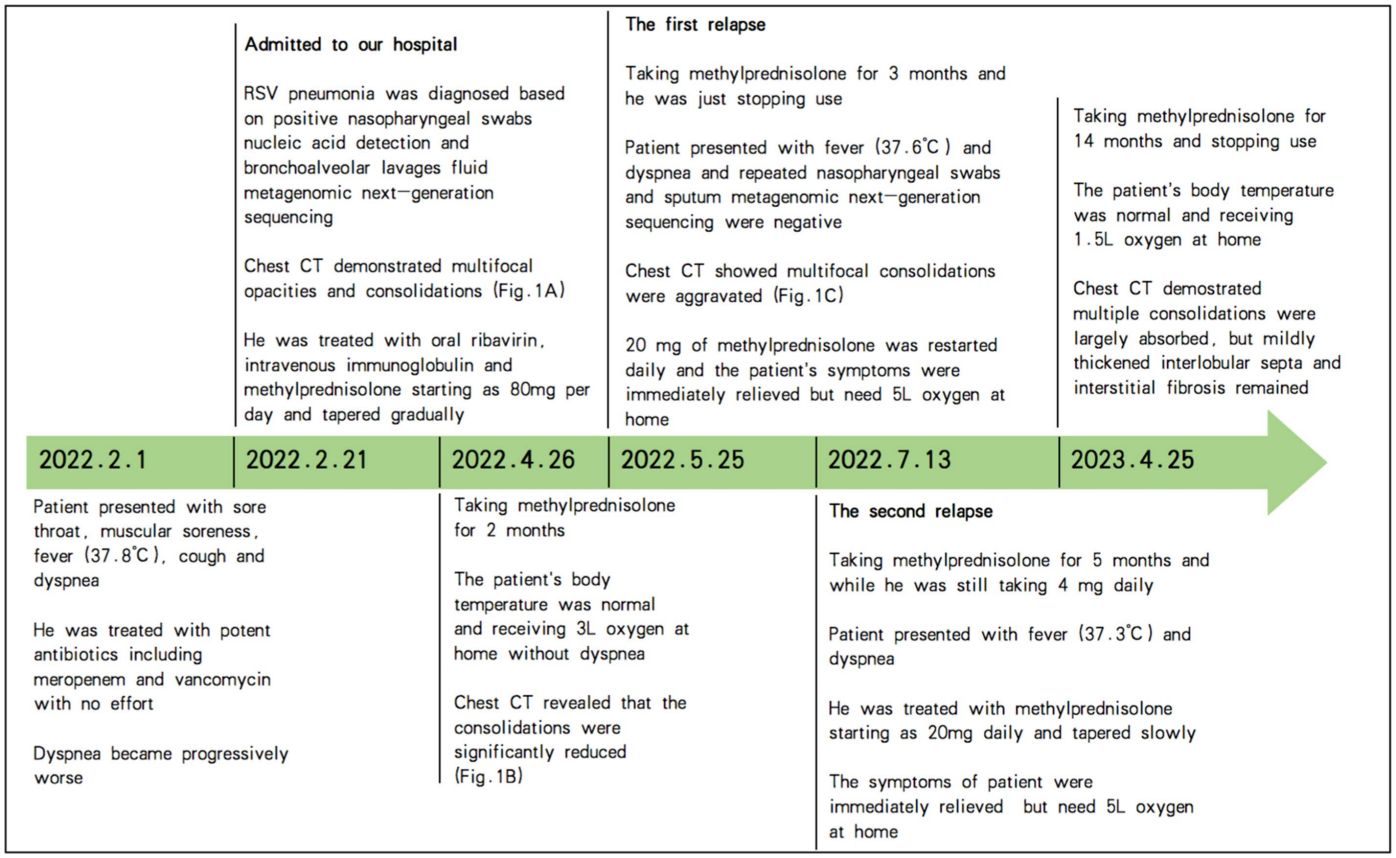
Timeline of the patient’s diagnosis, treatment and follow-up over a 14-month period. RSV, respiratory syncytial virus; CT, computed tomography; Fig., figure.

However, the patient experienced two relapses during corticosteroid treatment, one after taking methylprednisolone for 3 months and while he was just stopping use, and the other after taking methylprednisolone for 5 months but while he was still taking methylprednisolone 4 mg daily. The patient presented with a low-grade fever and dyspnea again. The chest image revealed that bilateral and multifocal opacities and consolidations were aggravated again (Figure 1C). Repeated nasopharyngeal swabs and sputum metagenomic next-generation sequencing were negative both times. Therefore, 20 mg of methylprednisolone was restarted daily, and the patient’s symptoms were immediately relieved. Figure 2 summarizes the timeline of the patient’s diagnosis, treatment and follow-up over a 14-month period. Despite the lack of pathological evidence because the patient refused transbronchial lung biopsy, we still diagnosed secondary organizing pneumonia after RSV infection based on the typical clinical manifestations and characteristics of this patient; therefore, the patient resumed taking 20 mg of methylprednisolone per day, and the dose was reduced slowly, with the entire course of therapy lasting for another 9 months in total. Re-examination of chest CT showed that the multiple consolidations in both lungs were largely absorbed, but mildly thickened interlobular septa and interstitial fibrosis remained. After stopping the corticosteroids, the patient has had no fever or dyspnea, and 1.5 L/min oxygen has been intermittently used at home.

## 3 Discussion

The patient in this case was an elderly individual over 65 years old who had a variety of chronic cardiopulmonary diseases. He showed clinical manifestations of viral pneumonia, such as sore throat, muscle aches, fever and cough. However, the symptoms of RSV are nonspecific, and infections with RSV cannot be distinguished from other viral infections (such as influenza virus). The patient experienced disease onset during the RSV epidemic season, and empirical antibacterial treatment outside the hospital was ineffective. The disease progressed rapidly, and severe respiratory failure occurred; as a result, he was admitted to our respiratory intensive care unit (ICU). RSV was detected by PCR of nasopharyngeal swabs and metagenomic next-generation sequencing of BALF, so severe RSV pneumonia was diagnosed. At present, there are no guidelines for the management of RSV pneumonia in adults. Aerosolized ribavirin was recommended by the US Food and Drug Administration (FDA) for the treatment of RSV infection in infants and young children. Now, there have been many reports on the use of oral or intravenous ribavirin as an alternative treatment for RSV infection (19–21). In addition to ribavirin, the treatment of RSV infection also includes the application of immunoglobulin and corticosteroids (22). Yoon et al. (23) reported the case of an 81-year-old previously healthy man who was diagnosed with RSV pneumonia complicated with acute respiratory distress syndrome. With the support of mechanical ventilation, oral ribavirin and low-dose steroids were given, and the prognosis was good.

We diagnosed this patient with RSV-related SOP mainly based on typical clinical manifestations, imaging features, corticosteroid therapy response and long-term follow-up. First, pulmonary infection is the most common cause of SOP (10). In this case, the patient was diagnosed with severe RSV pneumonia accompanied by respiratory failure, which led to the lung injury. Second, high-resolution chest CT showed typical imaging manifestations of OP: consolidation of both lungs mainly along bronchial vascular bundles and subpleural distribution, accompanied by bronchial meteorology, which can be observed in 79–95% of OP patients (24–26). It has been reported that the diagnostic rate of typical high-resolution chest CT for OP can reach 79% (27). Third, the disease response of the patient to steroid therapy was excellent, which is a prominent feature of OP (28). Two rounds of steroid tapering led to disease relapse of our patient, accompanied by flu-like symptoms such as fever and dyspnea, but the resumption of 20 mg of methylprednisolone quickly relieved the patient’s symptoms. It has been reported that recurrent OP can be quickly relieved if more than 20 mg of steroids are administered. Moreover, the rate of single and multiple (average 2.4 times) relapses of OP is 13–58%, and relapses usually occur when steroids are stopped or if the steroid dose is reduced to less than 20 mg/day (29). There are various risk factors for OP recurrence, including a delay from the first symptom to treatment; extensive consolidation of both lungs, involving the upper, middle and lower lobes; severe hypoxemia; high pathological fiber deposition; a high neutrophil ratio in bronchial lavage fluid; and high KL-6 (30–33). This patient had many concurrent risk factors for OP recurrence. Fourth, during the 14-month follow-up, repeated chest CT and laboratory tests did not suggest other potential diseases, such as tumors or connective tissue diseases. Finally, the inflammation in both lungs was basically absorbed, but a small amount of the interlobular septal thickening and mild fibrosis remained. According to the literature, approximately 70% of patients can have residual lung lesions after steroid therapy, and severe consolidation, a long treatment period and bronchiectasis are risk factors leading to residual interstitial lung fibrosis (34, 35). At present, there are several classic schemes for tapering steroids in OP patients, with a total course of treatment of 6–12 months (36–38). However, some studies have shown that the recurrence of OP does not affect patient prognosis. Generally, adding corticosteroids at a dose of 20 mg and gradually reducing the dose can effectively control the disease. In view of the side effects of long-term use of corticosteroids, therapy can be shortened to 3 months after eliminating the cause of SOP, and extending the course of corticosteroid therapy should be considered for patients who experience relapse after reduction (39).

In this case, the patient’s initial onset was accompanied by severe respiratory failure, and the risk associated with lung biopsy was extremely high. Pneumothorax, bleeding or infection could have further aggravated the patient’s hypoxemia and could have even been fatal. Because this patient did not agree with invasive procedures such as transbronchial lung biopsy, this case lacked a pathological basis for diagnosing OP. At present, there are many case reports in which SOP was diagnosed in the absence of a pathological basis (40–43). Some studies point out that the pathological diagnosis of OP is mainly used to exclude other potential causes, and the presence of typical imaging manifestations combined with evaluating the medical history, utilizing steroid therapy and keeping close follow-up can avoid unnecessary biopsies (38, 44–46). We believe that invasive lung biopsy and pathological examination should be carried out after fully weighing the advantages and disadvantages of these methods, especially for critically ill patients with severe respiratory failure. The potential complications of lung biopsy may be fatal. We recommend long-term and close follow-up to exclude other diseases for patients with high clinical suspicion of SOP for whom pathological evidence cannot be obtained.

## 4 Conclusion

RSV has gradually become an important pathogen of respiratory tract infections in elderly people with underlying diseases. Physicians should further enhance their awareness of the clinical manifestations and management of RSV pneumonia. During the epidemic period of RSV, it is recommended that pathogenic tests, including those for RSV, be performed to make an etiological diagnosis. For adult patients with severe RSV respiratory tract infections, the main drug treatments include aerosolized, oral or intravenous ribavirin, immunoglobulin and corticosteroid therapy, and standardized treatment guidelines still need further study. We report a patient with suspected SOP after severe RSV pneumonia who experienced two relapses during the process of corticosteroid tapering. Although this case lacked pathological evidence of OP, according to the typical imaging manifestations and the clinical characteristics of good response to corticosteroids and the course of 14 months of management and close follow-up, this patient was successfully treated.

## Data availability statement

The original contributions presented in the study are included in the article/supplementary material, further inquiries can be directed to the corresponding author.

## Ethics statement

The studies involving humans were approved by Ethics Committee of First Affiliated Hospital of Dalian Medical University. The studies were conducted in accordance with the local legislation and institutional requirements. The participants provided their written informed consent to participate in this study. Written informed consent was obtained from the individual(s) for the publication of any potentially identifiable images or data included in this article.

## Author contributions

MF: Writing – review & editing, Supervision, Validation. JZ: Data curation, Resources, Writing – original draft. XL: Writing – original draft, Data curation, Resources. SW: Writing – review & editing, Data curation, Resources, Software. YL: Writing – review & editing, Supervision, Validation. CD: Data curation, Resources, Writing – review & editing, Conceptualization, Methodology, Supervision, Validation, Writing – original draft.
